# Quality Assessment of Functional Claims of Salacia-Derived Salacinol-Containing Foods

**DOI:** 10.7759/cureus.73798

**Published:** 2024-11-16

**Authors:** Kazumi Kuribayashi, Takahiro Amemiya, Toshikazu Seino, Takashi Tomita

**Affiliations:** 1 Department of Pharmaceutical Sciences, Faculty of Pharmaceutical Sciences, Teikyo Heisei University, Nakano-ku, JPN; 2 Department of Pharmacy, Sanno Hospital, Minato-ku, JPN; 3 Department of Pharmaceutical Sciences, School of Pharmacy at Narita, International University of Health and Welfare, Narita-shi, JPN; 4 Department of Pharmacy, International University of Health and Welfare, Mita Hospital, Minato-ku, JPN

**Keywords:** disintegration test, functional claims food, japanese pharmacopoeia, salacia derived salacinol, weight variation test

## Abstract

Background

Functional claim-associated foods contain raw materials that contribute to enhancing health in Japan. However, certain foods with functional claims fail to meet the disintegration test (DT) criteria of the Japanese Pharmacopoeia (JP).

Objective

This study assessed the quality of tablet-type, functional claim-associated foods containing Salacia-derived salacinol, using DT and weight variation (WV) tests.

Methods

DT and WV were conducted on functional foods (A, B, and C) sourced from three companies using samples stored either at room temperature (RT: 22-27 ℃) or in an incubator at 40 ± 2 °C and 75% ± 5% in relative humidity accelerated conditions (AC) for one, two, and four weeks, respectively.

Results

Samples A and C were compliant under all WV test conditions, whereas sample B was compliant except when stored in AC. DT results indicated compliance for sample A upon immediate opening and on storing for two and four weeks at RT, sample B for four weeks at RT, and sample C upon immediate opening, all AC, and stored for two and four weeks at RT.

Conclusions

Varied disintegration times may affect the release of functionally active ingredients into the body and their absorption from the gastrointestinal tract, potentially compromising their intended health benefits.

## Introduction

Recently, lifestyle-related diseases have surged in Japan owing to the westernization of dietary habits and a worsening lack of exercise [1,2]. Approximately 18.5 million people suffer from seven major diseases: cancer, heart disease, cerebrovascular disease, diabetes, hypertension, chronic kidney disease, and liver cirrhosis, affecting approximately one in seven Japanese people [3]. Therefore, an increasing number of individuals are self-medicating by using healthy foods and supplements [4]. Among these, foods with functional claims, which are marketed under the responsibility of business operators, are useful for maintaining and promoting health [5]. The business operator issuing the notification should ensure the conformity of scientific basis and labeling content. Additionally, they should explain the scientific basis for safety and functionality [5]. In practice, clinical trials and systematic reviews are used to assess the scientific evidence regarding functionality [5].

Standards for pharmaceutical products are established based on good manufacturing practices (GMP) to ensure a certain level of quality [6]. However, functional claims for foods are notified to the Commissioner of the Consumer Affairs Agency, enabling business operators to assert specific health benefits based on scientific evidence regarding safety and functionality without individual review [7]. A study based on the Japanese Pharmacopoeia (JP) assessed the disintegration of food products in tablet and capsule forms, and the results demonstrated that 42 of 100 products did not disintegrate within the specified time [8]. This quality concern indicates that specific health objectives may not be achieved. We thus considered it important to first evaluate the quality of the product based on DT. The purpose of this study was to evaluate the quality of a tablet-type functional food in bottle packaging containing Salacia-derived salacinol, which inhibits the absorption of sugar, using a DT [9,10]. In addition, a weight deviation (WV) test was conducted to examine whether weight change occurred due to humidity.

## Materials and methods

We used samples A (Lot:AI), B (Lot:JHB), and C (Lot:901) from three companies whose products are commercially available in plastic bottles and have been recognized by the Consumer Affairs Agency as functional claim-associated foods containing Salacia-derived salacinol. However, only sample C used during the one-week storage belonged to a different product lot (Lot:002). Each sample was sealed in a zippered polyethylene bag (Unipack, SEISANNIPPONSHA LTD., Tokyo, Japan) and stored either at room temperature (RT: 22-27 ℃) or in an incubator (LHL114; ESPEC CORP., Osaka, Japan) at 40 ± 2 °C and 75% ± 5% relative humidity under accelerated conditions (AC) for one, two, and four weeks, respectively.

Weight variation (WV) test

Ten random samples from A, B, and C were accurately weighed using an analytical balance (AUY120; Shimazu Corporation, Kyoto, Japan) immediately after opening, under AC, and RT for each storage period, following the JP13 WV test protocol [11]. Subsequently, mean weight, standard deviation (SD), and relative standard deviation (RSD) were calculated. When the judgment value in 10 pieces did not exceed 15.0%, the following formula was used to determine conformity:

Judgment value = |M-A|+kS 

M: The indicated amount (100.0%) was used unless otherwise specified.

A: Average content per sample was determined using a quantitative method (% of the labeled amount).

k: Determination coefficient

When n is 10, k = 2.2.

S: SD of samples

The specified values for each product were 603 mg/three grains (0.201 g/grain) for A, 1.8 g/six grains (0.3 g/grain) for B, and 1.8 g/three grains (0.6 g/grain) for C.

In this study, after precisely weighing each of the 10 pieces and calculating the judgment value, any piece with a value not exceeding 15.0% was considered compliant.

Disintegration test (DT)

Eighteen pieces each of A, B, and C, which were stored either immediately upon opening or for one, two, and four weeks under AC and RT conditions, respectively, were placed in glass tubes. The DT was conducted using a disintegration tester (NT-40HS, Toyama Sangyo Co., Ltd., Osaka, Japan) that moved back and forth (29-32 times/min) and up and down with an amplitude of 53-57 mm. Water was added at 37 ± 2 °C as the test solution to a glass tube following the JP18 DT method [12]. Samples were observed every 5 min for a total test time of 60 min for coated tablets (A) and 30 min for uncoated tablets (B and C). A sample was judged to have disintegrated when no residue of the sample was found in the glass tube, or if when it was found, it was a soft substance that clearly did not retain its original form or it was a fragment of an insoluble agent skin or capsule coating. A sample was considered compliant if more than 16 of the 18 samples collapsed. Eighteen pieces of each sample were tested (three vessels with six pieces each).

## Results

Weight variation (WV) test-based determination of suitability

The WV test was conducted to evaluate the weight of the product after it had absorbed moisture. Individual weights were measured for 10 pieces of each product obtained from the three companies. The WV test results are presented in Table 1.

**Table 1:** Results of the weight variation test Ten samples were randomly selected from A, B, and C. AC: accelerated conditions (40 ± 2 °C, 75% ± 5% relative humidity), RT: room temperature, N.P.: not possible, SD: standard deviation, RSD: relative standard deviation

Product	Storage conditions	Period (week)	Average weight (g) ± SD before/after		RSD (%) before/after		Weight difference (g)	Judgment value (%) before/after	
A		0	0.195±0.002		1.13			2.80	
	AC	1	0.202±0.004	0.219±0.004	1.83	1.84	+0.017	0.208	9.01
		2	0.198±0.001	0.218±0.001	0.650	0.559	+0.02	1.50	8.50
		4	0.198±0.002	0.220±0.002	0.923	0.906	+0.022	1.50	9.40
	RT	1	0.201±0.003	0.201±0.003	1.45	1.45	0.00	0.51	0.51
		2	0.198±0.002	0.198±0.002	0.774	0.825	0.00	0.51	0.006
		4	0.198±0.001	0.198±0.002	0.681	0.787	0.00	1.50	1.50
B		0	0.299±0.002		0.668			0.304	
	AC	1	0.301±0.002	N.P.	0.700	N.P.	N.P.	N.P.	N.P.
		2	0.300±0.002	N.P.	0.665	N.P.	N.P.	N.P.	N.P.
		4	0.301±0.002	N.P.	0.771	N.P.	N.P.	N.P.	N.P.
	RT	1	0.299±0.002	0.299±0.002	0.521	0.560	0.00	0.303	0.304
		2	0.301±0.002	0.301±0.002	0.780	0.776	0.00	0.305	0.205
		4	0.300±0.002	0.304±0.008	0.673	2.71	+0.003	0.004	1.32
C		0	0.302±0.002		0.545			0.704	
	AC	1	0.299±0.001	0.324±0.001	0.453	0.422	+0.025	0.303	8.00
		2	0.301±0.002	0.323±0.002	0.564	0.537	+0.022	0.304	7.70
		4	0.301±0.002	0.322±0.002	0.562	0.563	+0.021	0.304	7.30
	RT	1	0.300±0.001	0.301±0.001	0.302	0.192	+0.001	0.002	0.301
		2	0.301±0.002	0.301±0.002	0.549	0.568	0.00	0.304	0.303
		4	0.302±0.002	0.304±0.002	0.518	0.551	+0.002	0.703	1.30

**Figure 1:**
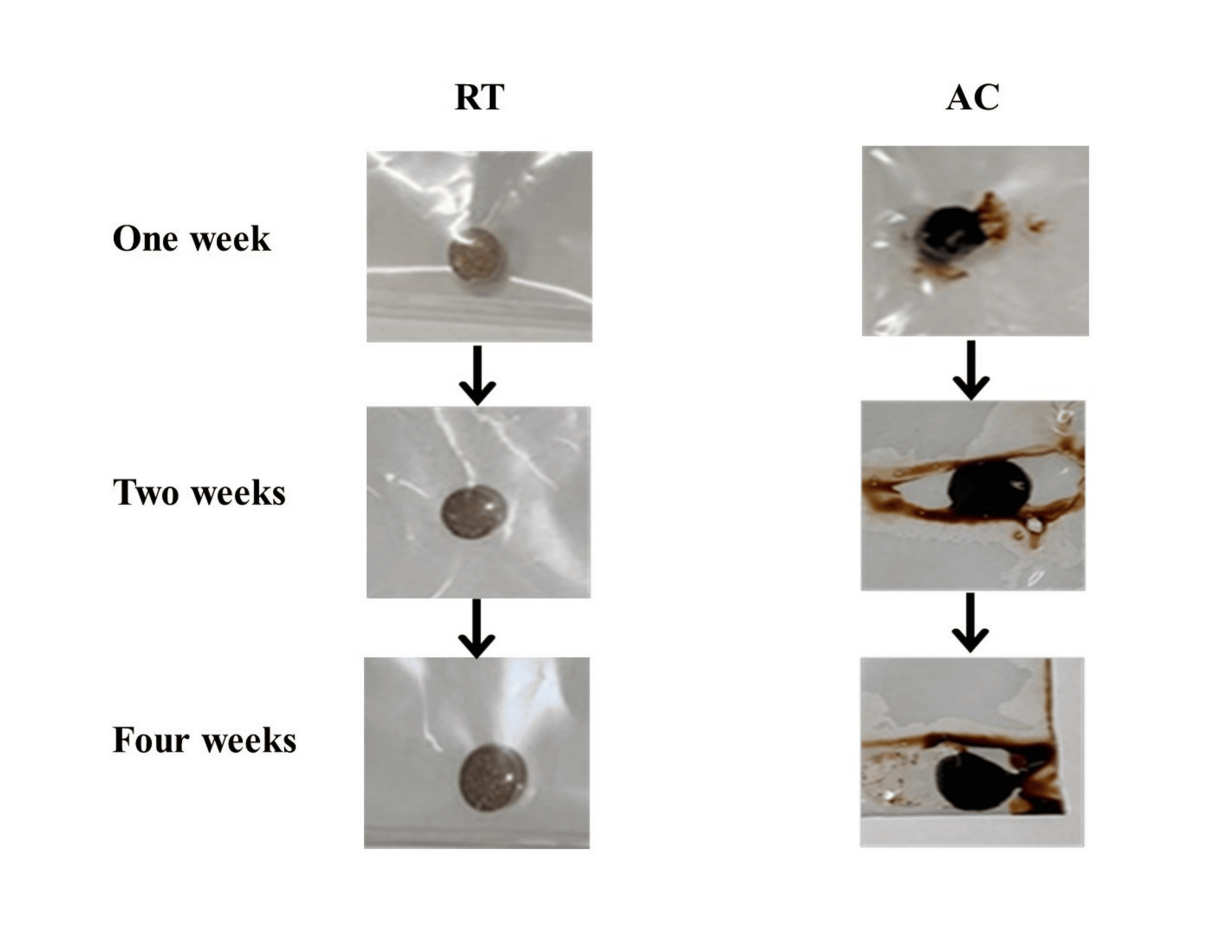
Alterations in the state of B during AC storage for a specific period RT: room temperature, AC: accelerated conditions (40 ± 2 °C, 75% ± 5% relative humidity)

The WV test was not conducted on sample B, which had been stored in AC for a while, because the sample was wet and adhered to the Unipack, making it difficult to remove (Figure 1).

Initially, all products complied with the test values of 15.0% or less immediately upon opening. The RT and AC values of the samples after each storage period, except for sample B (stored in AC), were both less than 15.0% and compliant.

Disintegration test-based determination of suitability

The DT results immediately on opening the package and after storing the samples for a certain period under AC or RT are summarized in Table 2. 

**Table 2:** Results of the disintegration test A sample was judged to have disintegrated when no residue of the sample was found in the glass tube, or if when it was found, it was a soft substance that clearly did not retain its original form or it was a fragment of an insoluble agent skin or capsule coating. A sample was considered compliant if more than 16 of the 18 samples were collapsed. AC: accelerated conditions (40 ± 2 °C, 75% ± 5% relative humidity), RT: room temperature

Product	Dosage form (test time)	Storage conditions	Period (week)	Judgment	Total number of collapses (pieces)
A	Coating agent (60 min)		0	Compliant	16
		AC	1		3
			2		3
			4		1
		RT	1		12
			2	Compliant	16
			4	Compliant	16
B	Uncoated tablet (30 min)		0		13
		AC	1		
			2		
			4		
		RT	1		15
			2		14
			4	Compliant	17
C	Uncoated tablet (30 min)		0	Compliant	18
		AC	1	Compliant	17
			2	Compliant	18
			4	Compliant	18
		RT	1		13
			2	Compliant	18
			4	Compliant	18

Sixteen pieces of sample A collapsed immediately upon opening and were compliant. Twelve disintegrations at one week resulted in non-compliance; however, 16 disintegrations at two and four weeks resulted in conformance at RT for sample A. In contrast, under AC for sample A, three disintegrations were observed at one and two weeks and one at four weeks, all of which were non-compliant. Despite the peeling of the coating, the tablets maintained their shape and size even after 60 min, with a minimal reduction in thickness. Additionally, the cumulative disintegration count was significantly lower for AC than for RT.

Thirteen pieces of sample B collapsed immediately upon opening and were non-compliant. For sample B, under RT, 15 disintegrated at one week and 14 at two weeks, and were non-compliant. In contrast, 17 pieces disintegrated at four weeks and were compliant. Additionally, DT was not conducted because the samples were blackened, muddy, and wet under AC, which adhered to the Unipack, making removing them difficult. Therefore, it was confirmed that sample B underwent alterations in shape when stored under hot and humid conditions for a certain period, similar to those in AC.

All pieces of sample C disintegrated immediately upon opening and were compliant. Under RT, 13 pieces disintegrated at one week and were non-compliant. However, all pieces disintegrated at two and four weeks and were compliant. Similarly, under AC, 17 pieces disintegrated at one week while all disintegrated at two and four weeks and were compliant.

## Discussion

In this study, salacinol was selected among a number of tablet-type functional claim-associated foods because its action is similar to that of drugs, many varieties are available, and it can be purchased at drugstores. To ensure the functional efficacy of healthy food products, it is necessary to conduct a DT to assess product disintegration in the body post-ingestion. WV and DT assessments of tablet-type functional foods in bottle packaging containing Salacia-derived salacinol revealed numerous products that were non-compliant in the DT. There are no established standards for manufacturing control and no clear methods for lot control in healthy foods. Additionally, the lack of guidelines for disintegration following a storage test that considers shelf life indicates a certain level of uncertainty in quality assurance. Consequently, it may affect the release of functionally active ingredients and their excretion from the body, thus potentially hindering the achievement of specific health benefits.

Despite belonging to the same lot, samples A (stored at RT for one week) and B (stored at RT immediately upon opening) exhibited variations in disintegration, resulting in non-compliance with the DT. DT has been conducted for functional foods in the form of supplements containing piperine and monoglucosyl hesperidin as functional ingredients, and it was discovered that certain products were non-compliant [13]. It is assumed that DT is conducted for each lot during the manufacturing of tablets and capsules. However, the lack of test method details and storage stability tests during the shelf life indicated that the disintegration property specified in JP is not maintained owing to poor packaging or product deterioration. Consequently, variations in actual quality may occur because the manufacturing control of healthy foods is a voluntary initiative by business operators.

DT was conducted on the samples stored in AC. The cumulative disintegration count in A was extremely low and all were non-compliant. Additionally, the shape of sample B was altered, which hindered DT. Therefore, this study indicated that quality deterioration may be because of storage under hot and humid conditions for a certain period.

No significant difference was observed in disintegration between AC and RT for sample C. However, the sample stored for one week at RT was non-compliant. This may be because DT was conducted on a different product lot during sample preparation for a one-week storage period.

This study selected functional foods (A, B, and C) because they are all packaged in bottles without individual press-through-package (PTP) sheets or other means. In Japan, summer temperatures can exceed 30 °C, and humidity may reach 80% during the rainy season, such as in July, according to the Japan Meteorological Agency [14]. Because of significant weather fluctuations throughout the year in Japan, proper storage of foods and supplements is crucial for quality control.

The study was limited to only three of the Salacia-derived salacinol-containing foods and was validated. The disintegratability of other functional claims food has not been verified and needs to be verified in the future. In addition, we were unable to find specific information on salacinol content in this study. Furthermore, this study focused on changes in disintegration due to humidity absorption and did not consider other aspects.

Based on the results of this study, we believe that the quality control of healthy foods should consider their disintegration and establish appropriate standards similar to those for pharmaceuticals to ensure consumer safety. While a relief system for adverse drug reactions is in place for drugs, no such system currently exists for healthy foods [15]. Interactions with pharmaceuticals are challenging examples of the serious health issues caused by healthy foods [16]. Therefore, care should be taken when combining healthy foods and pharmaceuticals because the efficacy of the pharmaceuticals may not be adequately demonstrated or adverse effects may become more prevalent [16].

## Conclusions

Manufacturing management and safety standards for healthy foods should be revised to safeguard public health. Additionally, it is crucial to offer data regarding the proper management and use of healthy foods and supplements. Upon opening, they should be stored away from high temperatures and humidity and consumed promptly, regardless of their expiration date.

## References

[1] Yotsumoto H, Kaneko H, Itoh H (2021). Promoting analysis of real-world data: prospects for preventive cardiology in Japan. Glob Health Med.

[2] (2024). Japan: universal health care at 50 years. https://www.thelancet.com/series/japan.

[3] (2024). Ministry of Health, Labor and Welfare. Patient survey [In Japanese]. https://www.mhlw.go.jp/toukei/saikin/hw/kanja/17/dl/01.pdf.

[4] Masumoto S, Nakayama G, Haruta J, Maeno T (2023). Association between experience of interprofessional care and self-medication among family caregivers: a cross-sectional study. Res Social Adm Pharm.

[5] Maeda-Yamamoto M (2017). Development of functional agricultural products and use of a new health claim system in Japan. Trends Food Sci Technol.

[6] Kajiwara E, Kamizato H, Shikano M (2020). Impact of quality by design development on the review period of new drug approval and product quality in Japan. Ther Innov Regul Sci.

[7] Kamioka H (2023). Current status and issues on the foods with function claims system in Japan: evidence of functionality of the foods [Article in Japanese]. Yakugaku Zasshi.

[8] (2024). Japan Health and Nutrition Food Association. Mandatory disintegration testing of tablets, capsules and other food products for GMP certified factories and the importance of confirming the content of active ingredients [Article in Japanese]. https://www.jhnfa.org/news-0239.html.

[9] Stohs SJ, Ray S (2015). Anti-diabetic and anti-hyperlipidemic effects and safety of Salacia reticulata and related species. Phytother Res.

[10] Chavan J, Patil P, Patil A (2024). Salacia spp.: recent insights on biotechnological interventions and future perspectives. Appl Microbiol Biotechnol.

[11] Ministry of Health, Labor and Welfare (1996). The Japanese Pharmacopoeia, Thirteenth Edition. https://www.amazon.com/Japanese-pharmacopoeia-XIII-official-April/dp/4840803897.

[12] Ministry of Health, Labor and Welfare (2021). The Japanese Pharmacopoeia, Eighteenth Edition. https://www.pmda.go.jp/english/rs-sb-std/standards-development/jp/0029.html.

[13] Masada S, Mizuno S, Kotani A (2019). Studies of physical quality evaluation of “foods with functional claims” containing piperine and monoglucosyl hesperidin as functional substances [Article in Japanese]. Jpn J Food Chem Saf.

[14] Zhang Y, Li S (2019). Climatological characteristics of planetary boundary layer height over Japan. Int J Climatol.

[15] (2024). Pharmaceuticals and Medical Devices Agency. Medicines adverse reaction relief system [In Japanese]. https://www.pmda.go.jp/kenkouhigai_camp/.

[16] Mouly S, Lloret-Linares C, Sellier PO, Sene D, Bergmann JF (2017). Is the clinical relevance of drug-food and drug-herb interactions limited to grapefruit juice and Saint-John's Wort?. Pharmacol Res.

